# Comprehensive genome-scale metabolic model of the human pathogen *Cryptococcus neoformans*: A platform for understanding pathogen metabolism and identifying new drug targets

**DOI:** 10.3389/fbinf.2023.1121409

**Published:** 2023-01-13

**Authors:** Enes Fahri Tezcan, Yigit Demirtas, Zeynep Petek Cakar, Kutlu O. Ulgen

**Affiliations:** ^1^ Department of Molecular Biology and Genetics, Istanbul Technical University, Istanbul, Turkey; ^2^ Department of Chemical Engineering, Bogazici University, Istanbul, Turkey

**Keywords:** *cryptococcus neoformans*, genome scale metabolic model, drug target, gene essentiality, metabolic reconstruction

## Abstract

**Introduction:** The fungal priority pathogen *Cryptococcus neoformans* causes cryptococcal meningoencephalitis in immunocompromised individuals and leads to hundreds of thousands of deaths per year. The undesirable side effects of existing treatments, the need for long application times to prevent the disease from recurring, the lack of resources for these treatment methods to spread over all continents necessitate the search for new treatment methods.

**Methods:** Genome-scale models have been shown to be valuable in studying the metabolism of many organisms. Here we present the first genome-scale metabolic model for *C. neoformans*, iCryptococcus. This comprehensive model consists of 1,270 reactions, 1,143 metabolites, 649 genes, and eight compartments. The model was validated, proving accurate when predicting the capability of utilizing different carbon and nitrogen sources and growth rate in comparison to experimental data.

**Results and Discussion:** The compatibility of the *in silico* Cryptococcus metabolism under infection conditions was assessed. The steroid and amino acid metabolisms found in the essentiality analyses have the potential to be drug targets for the therapeutic strategies to be developed against Cryptococcus species. iCryptococcus model can be applied to explore new targets for antifungal drugs along with essential gene, metabolite and reaction analyses and provides a promising platform for elucidation of pathogen metabolism.

## 1 Introduction

Human pathogens are organisms that cause disease in both healthy and immunocompromised individuals. *Cryptococcus neoformans* is a fungal pathogen that causes cryptococcosis in humans. In healthy people, *C. neoformans* is easily eliminated and rarely causes disease. However, it brings about cryptococcal meningoencephalitis, which causes 6,00,000 deaths annually in immunocompromised individuals (Chayakulkeeree and Perfect., 2008; Park et al., 2009; Dambuza et al., 2018). Cryptococcal meningoencephalitis has a major impact on AIDS and immunocompromised individuals, and once the infectious particles are inhaled, pulmonary infection occurs and can spread causing fatal meningoenceophalitis. This infection leads to death of about 20% of affected individuals. Because of this characteristics, *C. neoformans* is known as an opportunistic pathogen (Chayakulkeeree and Perfect., 2008; Dambuza et al., 2018; Park et al., 2009; Wang et al., 2018). There are different pathogenic *C. neoformans* isolates, of which serogroups A and D cause the cryptococcal meningoencephalitis in immunocompromised patients, whereas B and C affect the immunocompetent individuals. *C. neoformans* is acquired by inhalation from the environment in the form of spores or dry yeast, and in the early post-infection stage, colonization begins in lung, where the immune system response is provided by macrophages. However, if the infection is not cleared in the lung, it disseminates to the bloodstream and can affect major organs and systems. As a result of this dissemination of the pathogen to the brain, fatal cryptococcal meningoencephalitis occurs (Price et al., 2011; Rhome et al., 2011; Sabiiti and May., 2012; Waterman et al., 2012).

*C. neoformans* is a unique organism among other fungal pathogens with its cell wall and capsule structure, both are essential structural elements of the cell but are also key in its virulence. The composition of these structures changes dynamically, depending on the infected tissue. In addition, the fact that the synthesis stage of both the capsule and the cell wall, which are mainly composed of glycoconjugates, has not been determined in detail, constitutes an important obstacle for treatment options. (Mukaremera et al., 2018; Wang et al., 2018). There are three types of drugs that are widely used against *C. neoformans* infection (McEvoy et al., 2020; Mota Fernandes and Del Poeta., 2020). One of them, amphotericin B, is a drug in the polyene group and targets steroid metabolism (Gray et al., 2012; Anderson et al., 2014). Another antifungal compound that affects steroid metabolism is fluconazole, which is in the azole group. Caspofungin, on the other hand, is an echinocandin group compound and aims to inhibit the cell wall (McEvoy et al., 2020). However, current therapies have undesirable side effects and serious toxicity problems; long application times are required to prevent disease relapse, and due to a lack of resources these treatment methods cannot be equally spread to all continents. The drug Amphotericin B deoxycholate is nephrotoxic, resulting in the rising of creatinine and potassium and the wasting of magnesium within days of starting treatment. AmBd-induced anemia is another significant and less well-recognized adverse effect, resulting in a drop in hemoglobin from a baseline of 2–3 g/dL. The alternative antifungal drug Flucytosine (known as 5 FC) is very expensive and not licensed in most sub-Saharan African countries. It is also toxic to the body and in renal impairment, its levels should be monitored, and its dose should be adjusted as the drug accumulation increases the risk of bone marrow toxicity. Due to these adverse and side effects of all the existing treatment options, patients should have regular monitoring of complete blood count, electrolytes, and renal function (Perfect et al., 2010; Whitney and Bicanic, 2014; Denning, 2016). As a consequence, *C. neoformans* infections continue to be a problem worldwide, especially in developing countries, due to the high number of cases and deaths, healthcare and financial problems.

The development of novel therapeutics is complicated by many metabolic features that are shared between fungi and their human hosts. One of the major obstacles to the discovery of new drugs is the lack of general knowledge about the metabolism of organisms, especially during infection. This is exactly the case for *C. neoformans*, for which many studies focus on the potential antigen (Oliveira et al., 2021; Becerra-Álvarez et al., 2022; Nelson et al., 2022) and its functions and pathogenesis mechanisms (Sabiiti and May., 2012; Buchanan., 1998; Rhome et al., 2011; Waterman et al., 2012; Price et al., 2011; Mota Fernandes and Del Poeta., 2020; Gray et al., 2012; Anderson et al., 2014; Huang et al., 2016), and very few studies are carried out on the organism’s primary metabolism (Shea et al., 2006; Garcia-Santamarina et al., 2018; Berguson et al., 2022; Jezewski et al., 2022; Kinskovski and Staats, 2022; Ma et al., 2022). Even with genomic information, using genome-scale data to understand and interpret the function of genes in terms of the general biology of the pathogen remains challenging for systems-level understanding. However, the results of the analysis of the genomic data allow the reconstruction of the genome-scale metabolic reaction network. Genome-scale metabolic models (GSMMs) have been curated and converted to mathematical representation and studied to determine metabolic capabilities by using constraint-based methods in the computational systems biology approach (Kauffman et al., 2003; Beste et al., 2007; Orth et al., 2010). Constraint-based methods provide a useful framework for examining metabolism in different environmental conditions from a systematic perspective and understanding the responses of the complex biological network (Sun et al., 2009; Haggart et al., 2011). With the genome-scale metabolic models (GSMMs) created for pathogens, the basic metabolic characteristics of the organism, its virulence and interactions with the host can be elucidated (Dunphy and Papin., 2018; Sertbas and Ulgen., 2020). Through analyses with constraint-based simulation methods such as flux balance analysis on genome-scale models, the key reactions, metabolites and genes that are essential for maintaining the vital activities of the pathogen can be determined.

The need for developing new therapies by understanding the metabolism of this pathogen led us to reconstruct the genome-scale metabolic model specific to *C. neoformans* and to elucidate the metabolic processes of this pathogen using a computational systems biology approach. Potential drug targets were determined by examining how pathogen growth is affected by gene, reaction and metabolite inhibitions. This first metabolic model on *C. neoformans* will shed light on a better understanding the other *Cryptococcus* species, and may guide the fight against cryptococcus-caused infections and pathogenesis.

## 2 Materials and methods

### 2.1 Reconstruction of a draft model

The reconstruction of the genome-scale model of *Cryptococcus neoformans var. neoformans* JEC21 was performed in accordance with the protocol of Thiele and Palsson (Thiele and Palsson., 2010). The reactions in pathways were collected from the metabolic maps in the KEGG database (Loftus et al., 2005). The metabolites, genes and enzyme numbers for these reactions were collected from KEGG and CHEBI. CHEBI database (Degtyarenko et al., 2007) was used for the chemical formulas and charges of the metabolites. In order to determine the directions of the reactions collected from the KEGG database and ModelSEED database (Seaver et al., 2020) was used. All reactions collected in draft model reconstruction were written in BiGG Model notation (King et al., 2015).

One of the important steps in the draft model reconstruction is the determination of gene localization. This step is important for finding the compartment where the reactions take place. Thus, the metabolites to be produced and consumed in each compartment can be determined. For reactions whose compartments could not be determined based on these databases, the compartment in which the same reaction occurred in the iMM904 model developed for *S. cerevisiae* (Mo et al., 2009) and SpoMBEL1693 model developed for *Schizosaccharomyces pombe* (Sohn et al., 2012) was taken into account. Additionally, the localization of the genes collected from the KEGG database was searched in five different databases: Uniprot (Apweiler, 2004), Predator (Small et al., 2004), Panther (Mi et al., 2019), PredictProtein (Yachdav et al., 2014) and TargetP (Almagro Armenteros et al., 2019). The results from these five databases were compared and the compartments were determined. Gene–Protein-Reaction (GPR) associations were found for each reaction to determine the functions of genes and how they affect reactions.

The iCryptococcus model contains 8 compartments, 649 genes, 1,270 reactions, and 1,143 metabolites.

### 2.2 Refinement of the reconstructed model

#### 2.2.1 Adding reactions using literature evidence

In addition to the reactions in the KEGG database, reactions based on literature evidence were added to the model. Reactions for sphingolipid metabolism (Singh et al., 2017), cell wall formation (Wang et al., 2018) and capsule formation (Zaragoza et al., 2009; Casadevall et al., 2018) were obtained from literature and included in the model. Moreover, as the KEGG database does not have a metabolic map suitable for collecting reactions for oxidative phosphorylation for *Cryptococcus neoformans var. neoformans* JEC21, the oxidative phosphorylation pathway was taken from the iMM904 model (Mo et al., 2009) which is a manually curated model of the yeast *Saccharomyces cerevisiae,* a similar organism according to phylogenetic classification (Mo et al., 2009 **).**

#### 2.2.2 Adding spontaneous reactions

With the addition of spontaneous reactions in metabolic maps of the KEGG database, it is possible to reduce the number of dead-end metabolites and to ensure the connection of metabolites with the rest of the model (Thiele and Palsson., 2010). Spontaneous reactions with at least one metabolite linking to the rest of the remodeling were added to avoid too many dead-end metabolites caused by spontaneous reactions.

#### 2.2.3 Adding transport and exchange reactions

Since the model contains different compartments, metabolites must be transported between compartments. At the same time, metabolite exchange from/to the extracellular environment is important for the growth and reproduction of the organism. This is also essential for the prevention of metabolite accumulation in the cell. Therefore, transport and exchange reactions were included in the model.

#### 2.2.4 Manual gap-filing

There are several gaps in the metabolic network due to lack of information in the KEGG database as well as in the literature. This causes an increase in the number of dead-end metabolites in the model. Either some reactions do not take place due to the absence of reactants, or the produced metabolites cannot be consumed and accumulate in the cell. For this reason, manual gap-filling was applied to identify and fill gaps in the network.

In this process, dead-end metabolites in the *in silico* Cryptococcus model were first detected. The dead-end metabolite is the metabolite that is produced but not consumed in the model or not present in the model although it is a reactant in at least one reaction. In addition to dead-end metabolites, blocked reactions are essential for the gap-filling process. These reactions do not have flux due to the model topology and/or dead-end metabolites. Therefore, the blocked reactions in the model were detected after finding the dead-end metabolites. In the next step, new demand and sink reactions were proposed by taking the reactions in the literature, dead-end metabolites, and blocked reactions into consideration.

#### 2.2.5 Adding demand and sink reactions

In steady-state models, the demand reactions are reactions that allow the compound to accumulate and not be used due to mass balance requirements. For each metabolite, the sum of the inflow at steady state must equal to the sum of the outflow for mass balance requirements. Demand functions can only be added to the model for components that are known to be produced by the organism. Sink reactions are similar to demand reactions but are defined to be reversible.

Demand and sink reactions were added in order to activate the blocked reactions detected by the gap-filling process. In this way, metabolites, which should be in the organism but are not available in the genome data, have been added to the network.

#### 2.2.6 Adding biomass reaction

Biomass biosynthesis was set as a linear combination of the macromolecules protein, DNA, RNA, lipid, carbohydrate, cell wall and capsule, which were considered to account for the overall biomass structure (Baart et al., 2007)**.** The biomass reaction for *C. neoformans* was based on the iMM904 model developed for *S. cerevisiae.* Detailed information on the cell wall and capsule structure of *C. neoformans* was combined with the overall *S. cerevisiae* cell composition, and the biomass reaction was modified to reflect the *C. neoformans* cell. (Lesage and Bussey., 2006; Mukaremera et al., 2018). Glucose was used as the carbon source to calculate flux of biomass reactions. The glucose uptake flux was set to 4 mmol/gDW/h, the growth rate was calculated and the doubling time was determined. Non-growth-associated maintenance cost means the amount of energy required for the vital activities of the organism except growth. This maintenance cost could be taken as 5 mmol/gDW/h for pathogenic organism models (Henson et al., 2019). In the iCryptococcus model, the lower bound of ATP maintenance reaction (ATPM) was set to 5 mmol/gDW/h. If the amount of ATP produced by the carbon source remains below this value, the iCryptococcus model will not produce any solution.

#### 2.2.7 Quality control

Numerous tests have been carried out to check the key features of the constructed genome-scale model and to see if it is capable of predicting metabolic processes in the *C. neoformans* pathogen. MEMOTE is an online platform developed for evaluating the quality of reconstructed metabolic models (Lieven et al., 2020). The MEMOTE test was first used to determine information such as the consistency of the model, metabolites, reactions, GPR associations, and biomass (Lieven et al., 2020). Additional basic tests were then performed, including leakage, ATP demand, and energy production from water and/or oxygen properties (Heirendt et al., 2019).

Several properties of the reconstructed iCryptococcus metabolic model were first tested in MEMOTE platform, and the scores were assigned specifically to the tested features. The iCryptococcus model reached a score of 97% in the consistency section, including the categories of stoichiometric consistency, mass/charge balance, and metabolite connectivity. According to MEMOTE results, there were no duplicate reactions or metabolites in the iCryptococcus model. The metabolic coverage value of the iCryptococcus model was 1.96, as estimated by MEMOTE. This value indicates the level of modeling detail of the metabolic model. If the metabolic coverage is greater than 1, the model has a high level of modeling detail. The test on genes and reactions indicated that 193 reactions in the metabolic model did not contain GPR, and most of them were transport and exchange reactions. There are 47 enzyme complexes in the iCryptococcus model.

The biomass consistency was calculated as 0.95. The biomass value was 0.36/h for the default medium.

##### 2.2.7.1 Checking basic properties

The iCryptococcus model was checked for its functionality through the COBRA Toolbox (Becker et al., 2007), by following the sanity checks tutorial prepared by Thiele and colleagues. Two different leak checks were performed using the fastLeakTest function: i) test whether any metabolite was excreted under the condition that the uptake of any metabolite was not allowed; ii) check any leakage after adding the demand reaction for each metabolite in the iCryptococcus model. The result of both tests indicated no leakage in the iCryptococcus model.

In order to test whether the model generates energy from water and/or oxygen, the exchange reactions of all metabolites except water and oxygen were stopped. There was no energy production under these conditions, and the iCryptococcus model does not produce energy from water or oxygen. Similarly, it was tested whether there is any matter production when the lower bounds of the uptake reactions of all metabolites are set to zero and the ATP demand reaction is reversible. Fortunately, there was no matter production under these conditions. The demand reactions for h[c] and h[m] were added to control the proton production in the iCryptococcus model. Except for these reactions, the uptake of all exchange reactions was stopped. As a result of this test, the iCryptococcus model does not produce any proton from nothing.

ATP demand from the carbon source under aerobic conditions was also tested, where no constraint was applied to the water and oxygen exchange reactions. The ATP demand reaction was set as the objective function of the model. After applying flux balance analysis, the flux passing through the ATP demand reaction was compared with the threshold value (31 mmol/gDW/h in this test). The ATP demand from glucose was found not to exceed this threshold value in the iCryptococcus model, indicating no other reaction mechanism besides the oxidative phosphorylation is involved in ATP production.

### 2.3 Flux balance analysis

Constraint-based modeling is a widely used strategy to interpret the responses of organisms with complex metabolic networks under different conditions. This method, which is applied in genome-scale metabolic models and works under the assumption of a steady state, contributes to the elucidation of the genotype-phenotype relationship by using some features specific to the metabolic network of the organism as constraints. Flux balance analysis (FBA) examines the flow of metabolites in a metabolic network by means of network-specific constraints, and enables to predict the growth of the organism or the production of a particular metabolite according to the intended use of the organism. In FBA, the mass balance equation for each reaction and reaction reversibility are used as constraints. For the mathematical representation of the reactions in the metabolic network, a stoichiometric matrix (S) with the dimension (m x n) containing the stoichiometric coefficients of the metabolites in each reaction is used where m represents the metabolites and *n* represents the reactions. The values corresponding to a reaction column give the stoichiometric coefficients of the metabolites in that reaction. A negative stoichiometric coefficient is used for each metabolite consumed in a reaction, while a positive coefficient is used for metabolites produced. Fluxes are further subjected to capacity constraints, i.e., adding an upper bound and a lower bound for each reaction. In this study, the lower bounds of irreversible reactions were set to zero and the upper bound to +1,000. The lower limit of reversible reactions (430 reactions) was set to −1,000 and the upper limit to +1,000. These limits allow reactions to carry positive or negative flows under steady-state conditions in which there is no accumulation of intracellular metabolites.

In the optimization step, an objective function that satisfies all the constraints must be defined to find a single solution space. The objective function may be the growth of the organism, or it may be the flux through a reaction related to the production of a metabolite of interest. Linear programming was used to determine maximal theoretical flux with constraint (Kauffman et al., 2003; Orth et al., 2010). Due to the optimization technique based on the linear programming principle, a flux profile that maximizes the objective function of biomass production was obtained with iCryptococcus model.

### 2.4 Robustness analyses and shadow prices

Robustness analysis is applied to find the sensitivity of objective function of the model to a specific reaction (Orth et al., 2010). In this study, the effect of glucose uptake on the growth reaction was investigated by increasing its uptake values from 0–20 for two different conditions in which oxygen uptake was limited and unlimited. The optimal flux value for the objective function was calculated as a function of the altered flux through the glucose uptake reaction.

The shadow price of a metabolite in the model shows the sensitivity of the biomass objective function to the change in the amount of this metabolite (Orth et al., 2010). In other words, this value reveals how much the presence or absence of a metabolite will increase or decrease the target function, i.e., the objective function of biomass formation. The shadow prices of glucose and oxygen were calculated for each specific solution of the model. While determining the shadow price set of glucose, the lower and upper bounds of the oxygen uptake reaction were set to 4. Similarly, when calculating the shadow price set of oxygen, the lower and upper bounds of the glucose uptake reaction were set to 4.

### 2.5 Deletion analyses: Genes/reactions/metabolites

Identifying the genes that are essential for the growth and proliferation of a pathogen is important for drug strategies to be developed against the pathogen. Thus, it is possible to disrupt the metabolic activities of the pathogen and prevent its proliferation in the host. The use of computational analysis methods in the identification process of essential genes is significantly more time-saving than experimental methods. Single and double deletion studies were performed to find essential genes for *C. neoformans var. neoformans* JEC21. For this purpose, singleGeneDeletion and doubleGeneDeletion functions in the COBRA Toolbox (Becker et al., 2007) were used. The growth medium contained glucose as the carbon source.

In single-gene deletion analysis, the biomass production was calculated for each deleted gene and compared with the biomass production before the deletion. The genes were defined as essential when the ratio of biomass production with gene deletion to that of without the deletion was less than the threshold value of 1e^−3^.

The human orthologs of essential *Cryptococcus* genes were searched in Panther (Mi et al., 2019) and Inparanoid (Sonnhammer and Östlund, 2014) databases.

In the double-gene deletion analysis, the same procedure was applied with the deletion of two separate genes together. If the biomass production obtained as a result of deletion of these genes was less than the threshold value (1e^−3^), these genes were determined as an essential gene pair. The essential genes obtained in the single-gene deletion analysis were not used in this analysis, since they significantly reduce or stop the biomass production irrespective of the genes with which they were paired.

Metabolite deletion analysis was applied to identify metabolites essential for the metabolic activities of *C. neoformans*. For this purpose, EMFilter approach was used to filter metabolites in the iCryptococcus model (Kim et al., 2010). The metabolites involved in at least three reactions were filtered and the analysis was performed only for these filtered metabolites. It was aimed that the metabolite to be determined as the drug target will affect multiple reactions. For each of the metabolites involved in at least three reactions, the lower and upper bounds for reactions involving these metabolites were set to zero. Metabolites that make the biomass production zero due to the inhibiton of their reactions were determined. Currency metabolites (ATP, NAD, NADH, NADP, NADPH) were removed from these determined reactions, and the remaining metabolites were considered as essential metabolites. Similar to the gene deletion analysis, determining whether essential metabolites are present in the human model is important for the development of drug strategies without causing any side effects in humans. Thus, the metabolites identified as essential were compared with the metabolites in the human model (Recon3D).

In Reaction deletion analysis, the reactions necessary for the metabolic processes and growth were detected, similar to gene and metabolite deletion analyses. Thus, the principle of calculating the biomass production for each deleted reaction and comparing it with the biomass production before the deletion was used in the same way with the threshold value of 1e^−3^. For this purpose, the algorithm in the protocol published by Thiele and Palsson was used (Thiele and Palsson., 2010).

## 3 Results

### 3.1 Reconstruction of GEM for *Cryptococcus neoformans* var. *neoformans* JEC21

In the KEGG database, each metabolic pathway is represented by a metabolic map, which includes reaction and gene information obtained from the genome of the organism. In this way, 911 enzymatic reactions were collected from 57 pathways of *C. neoformans*, and the metabolites involved in these reactions, the gene(s) that regulate(s) each reaction, and the related enzyme numbers (EC number) were determined. There were 8 compartments in the model: cytosol, mitochondria, peroxisome, nucleus, endoplasmic reticulum, vacuole, extracellular space and golgi apparatus. Most of the reactions found in the model take place in the cytosol (660) and mitochondria (196). This is followed by peroxisome (28), nucleus (12), endoplasmic reticulum (6) vacuole (4), extracellular space (3) and Golgi apparatus (2), respectively.

The capsule and the cell wall are the important components of the organism observed in both *in vitro* and *in vivo* experiments. Therefore, in addition to the KEGG database, reactions for capsule, cell wall formation and sphingolipid metabolism were added to the model by using the evidence in the literature. Although the downstream steps of capsule synthesis have been elucidated in detail, the reactions involved in the final production phase from monomers have not yet been elucidated. Since the capsule is an important target component in drug studies, these reactions (without the catalyzing enzymes in the final synthesis step and the encoding genes) were included into the iCryptococcus model to complete the pathway as also done in many literature studies (Garcia et al., 2008; Singh et al., 2017; Wang et al., 2018).

Transport reactions between compartments were added to ensure the transfer of metabolites required for reactions taking place in different compartments in the cell. In this way, problems of accumulation of the metabolites or absence of reactants were eliminated. The metabolites to be taken into the cell or sent out of the cell were determined by literature search, the exchange reactions for these metabolites were added to the GSMM model. As a result, 239 transport reactions and 110 exchange reactions were added to the iCryptococcus model. In the manual gap-filling process, 11 spontaneous reactions in six pathways were added to reduce the number of dead-ends and five demand reactions were added to activate the blocked reactions in iCryptococcus model. The model also included one sink reaction.

**FIGURE 1:**
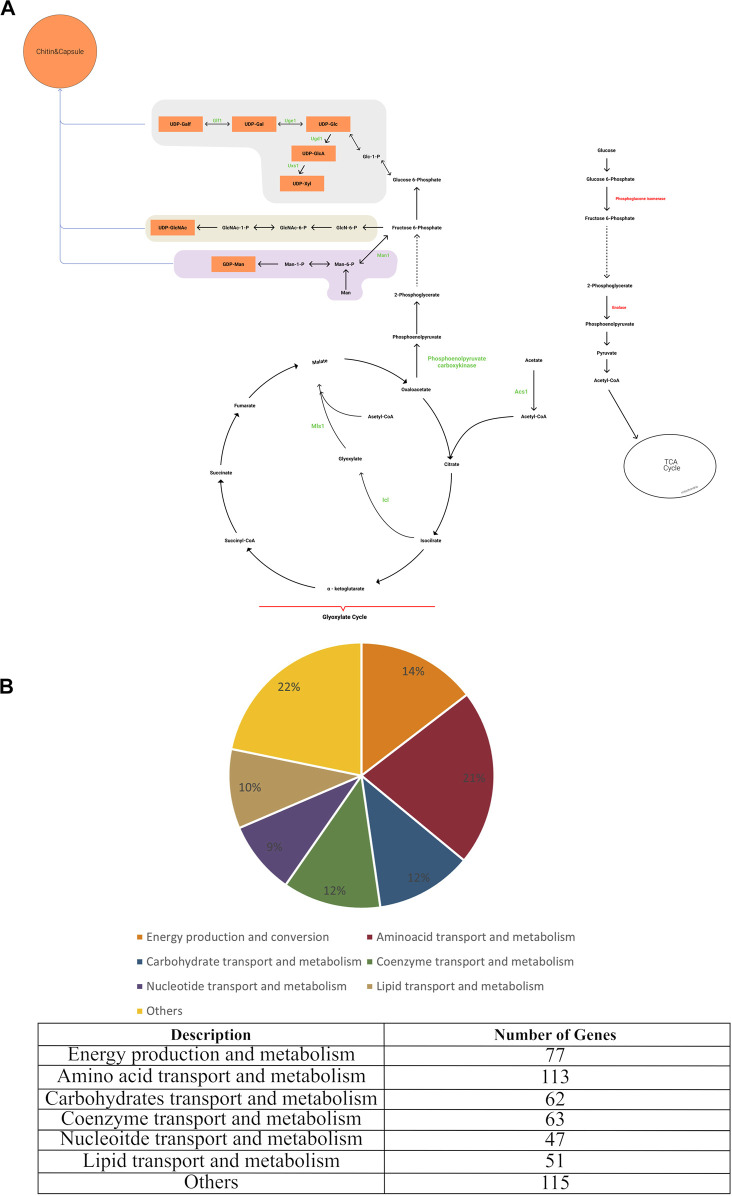
**(A)** Main metabolic reactions in iCryptococcus model including capsule formation, and **(B)** COG classification of *i*Cryptococcus model.

*C. neoformans* is a pathogen that is capable of growing in a host cell and also *in vitro*. Biomass composition data are available for *in vitro* grown yeast such as *S. cerevisiae* and these data were used as the basis for developing the biomass reaction for the *C. neoformans* cell *in silico* model. *C. neoformans* has a dynamic capsule and cell wall, unlike the yeast *S. cerevisiae*. In addition, many of the outer cell wall components of *C. neoformans*, although produced *in vitro*, are not essential for *in vitro* growth, but are required for pathogenesis (Wang et al., 2018). Structural analyses performed on samples grown under *in vitro* conditions revealed that the capsule was found as a thin layer surrounding the cell, and the cell wall was mainly composed of glucan. Ultrastructural studies of *C. neoformans* showed that the glucans in the cell wall are arranged in two layers. Compositional and imaging studies suggested that the inner layer consists of an alkali-insoluble meshwork of *ß*-glucan and chitin, while the less organized outer layer corresponds to an alkali-soluble fraction containing mainly *a*- and *ß*-glucans. Chitin and chitosan structures, which play an important role in pathogenicity, are present as minor components. Contrary to the results obtained from *in vitro* samples, it was concluded in the analyses made with *in vivo* samples that a thick capsular layer was formed, the main component of which was mannose, and that the chitin-chitosan structures in the cell wall increased proportionally (Mukaremera et al., 2018; Grossman and Casadevall, 2016; Zhou and Ballou, 2018; O’Meara and Alspaugh, 2012; Baker et al., 2007; Jang et al., 2022). In our study, to make the model applicable to *C*. *neoformans* grown both *in vitro* and *in vivo*, two biomass reactions based on experimentally derived values for macromolecular composition were defined. The first biomass reaction reflected the *in vivo* macromolecular composition of *C. neoformans*, and contained capsule structures such as mannose, xylose, and galactose essential for pathogenesis. The second biomass composition included only essential components for *in vitro* growth. Thus, the first biomass was employed in drug targeting studies and elucidation of gene and reaction pairs essential for virulence. The second biomass was used to make predictions regarding gene essentiality *in vitro*. With the completion of the gap-filling and adding the biomass reaction, the final model (*i*Cryptococcus) was obtained (Figure 1A).

#### 3.1.1 Information on metabolic reactions and their compartments

In the iCryptococcus model obtained, all reactions in the glycolysis/gluconeogenesis pathway took place in the cytosol with the product pyruvate which was then transported to the mitochondria where it was converted to acetyl-CoA. In the KEGG pathway map, this reaction was represented by the sum of four different reactions, R00014, R03270, R02569, and R07618. Instead of these four reactions, pyruvate dehydrogenase (PDHm) catalyzed reaction in the BiGG Model was taken as a single reaction during the metabolic model reconstruction. Similarly, the conversion of 2-oxoglutarate to succinyl-CoA in the TCA cycle was represented by the reactions R00621, R03316, R02570, R07618 in the KEGG database. Instead of these four reactions, 2-oxoglutarate dehydrogenase (AKGDm) catalyzed reaction in the BiGG Model was taken during the model reconstruction. In the starch and sucrose metabolic pathway, the conversion reactions between sucrose (sucr), D-fructose (fru), maltose (malt), D-glucose (glc D), D-glucose 6- phosphate (g6p), D-fructose 6-phosphate (f6p), trehalose (tre) and trehalose 6-phosphate (tre6p) take place. Glycogen (glycogen) and 1–3 beta D-glucan (13BDglcn) involved in the biomass reaction, were also produced in this pathway, and all these reactions took place in the cytosol.

In the pentose and glucuronate interconversion pathway, D-glucose 1-phosphate (g1p) is converted to UDP-glucose (udpg). In addition, some of the UDP-glucose is converted to UDP-glucuronate. While UDP-glucose is an important donor component for glucan in the cell wall structure, UDP-glucuronate acts as a donor for capsule structure components. All reactions in this pathway were included in the model and they took place in the cytosol. The amino sugar and nucleotide sugar metabolism pathway is of great importance as it enables crucial sugar donor production for capsule and cell wall production. The cell wall is composed of mainly glucan, and the other minor components are chitin and chitosan. UDP-glucose nucleotide sugar donor is needed for the synthesis of alpha and beta glucan structures. The sugar removed from the donor by various synthesis enzymes is added to the glucan structure. Chitin and chitosan are important components for growth and pathogenicity. Although chitin and chitosan have a minor contribution to the cell wall structure, their deficiency caused a decrease in virulence and a slowdown in the growth of the organism (Wang et al., 2018). Therefore, both components were involved in the biomass reaction. The capsule is an important component for *Cryptococcus neoformans* infectivity. Reduced virulence has been observed in non-encapsulated organisms in several studies (Chang and Kwon-Chung, 1994). Nucleotide sugars such as UDP-gal, UDP-glc, UDP-gala, UDP-xyl, and GDP-man involved in capsule formation are produced by this pathway.

The map of oxidative phosphorylation for Cryptococcus species was not provided in literature, including the KEGG database. Therefore, the oxidative phosphorylation pathway of the iMM904 model (Mo et al., 2009) developed for *S. cerevisiae* was used in the metabolic model reconstruction. The fatty acid degradation pathway takes place in the peroxisome wherein the fatty acids are broken down. Reactions starting from Palmitoyl-CoA (pmtcoa) continue until Acetyl-CoA (accoa) production. A lumped reaction (FAO80p in the BiGG Model) between octanoyl-CoA (occoa) and acetyl-CoA (accoa) was used during the GSMM model reconstruction.

Riboflavin (ribflv) is an important metabolite for cell growth as it is involved in the biomass reaction of the Cryptococcus model. In the riboflavin metabolic pathway, 3,4- dihydroxy-2-butanone 4-phosphate (db4p) is produced from D-ribulose 5-phosphate (ru5p_D). After the production of this metabolite, riboflavin (ribflv) is produced in the presence of 4- (1-D-Ribitylamino)-5-aminouracil (4r5au). Since there is no reaction for the production of 4- (1-D-Ribitylamino)-5-aminouracil (4r5au) in the KEGG database, this metabolite was added to the model as a demand reaction (DM 4r5au c).

The purine metabolic pathway is one of the pathways with the highest number of reactions in the model. Several currency metabolites are produced in this pathway and transported to different pathways. In terms of the metabolites produced and/or consumed, it is generally linked to the pyrimidine and thiamine pathways. In addition to the reactions in the KEGG map, the sink reaction for 5-amino-1-(5-phospho-D-ribosyl)imidazole-4-carboxylate (5aizc) and the demand reaction for (S)-2-[5-amino-1- (5-phospho-D-ribosyl)imidazole-4-carboxamido] succinate (25aics) were added to the model. These reactions generally take place in the cytosol.

In the terpenoid backbone biosynthetic pathway, metabolites having isoprene units are produced. Depending on the presence of (R)-5-phosphomevalonate (5pmev); (R)-5 diphosphomevalonate (5dpmev), isopentenyl diphosphate (ipdp), dimethylallyl diphosphate (dmpp), geranyl diphosphate (grdp) and farnesyl diphosphate (frdp) are produced. Farnesyl diphosphate, produced in this pathway, participates in the production of ergosterol in the steroid metabolic pathway. Since there is no reaction for the production of (R)-5-phosphomevalonate in the KEGG database, this metabolite was added to the model as a demand reaction (DM 5pmev c). The reactions in steroid biosynthesis pathway generally take place in the cytosol or endoplasmic reticulum. The organelles in which the reactions took place were determined using gene localization and the relevant transport reactions were added to the model. The steroid biosynthesis pathway is very important because of the variety of compounds produced, such as ergosterol. Besides being in the biomass reaction, ergosterol is also critical for the regulation of membrane fluidity (Farnoud et al., 2015; Rella et al., 2016). R00702 and R02872 reactions with the same EC numbers and genes in the pathway were combined in the model reconstruction process and written as squalene synthase (SQLS) catalyzed reaction.

#### 3.1.2 Clusters of orthologous groups of proteins (COG) classifications

In order to identify the categories of proteins encoded in the *C. neoformans* genome, the database of Clusters of Orthologous Groups of proteins (COG) (Tatusov., 2000) providing phylogenetic classification of proteins was used and the Gene IDs of the genes in the iCryptococcus model was searched. It was found that 21.4% of the genes of the iCryptococcus model were in the amino acid transport and metabolism category, followed by the categories of energy production and conversion, coenzyme transport and metabolism, and carbohydrate transport and metabolism by 14.58%, 11.93% and 11.74%, respectively (Figure 1B).

### 3.2 Validation studies of the iCryptococcus model

Available information on *Cryptococcus neoformans var. neoformans* JEC21 in the literature was used to simulate, evaluate and validate the iCryptococcus model. First, the growth rate obtained under MEMOTE default medium conditions was compared with experimental observations to validate the model. The doubling time of *C. neoformans* is between 80 min and 160 min, depending on the nutrient medium (Chen et al., 1997; Orner et al., 2019). The biomass production in the iCryptococcus model is 0.36/h in the default medium in MEMOTE. Based on this biomass production, the doubling time was calculated as 1.96 h (115 min), in line with the literature.

Phenotypic growth data were collected from several published literature to investigate *in silico* growth on different carbon and nitrogen sources. *C. neoformans* can grow on a variety of carbon sources (Barelle et al., 2006; Hu et al., 2008; Barkal et al., 2016). In the first simulation, we sequentially switched on the exchange reactions for the carbon sources and tested their performances for growth. This model correctly predicted the usability of various carbon sources such as glucose, fructose, acetate, fumarate, galactose, glycerol, sorbitol, succinate, lactate, ethanol, sucrose, lactic acid. Next, using glucose as the carbon source, the uptake fluxes of all nitrogen sources were sequentially turned on and off. If the on status resulted in an increase in biomass reaction flux, that nutrient was considered as growth-supporting one. In the literature, *C. neoformans* was reported to use all the amino acids, except for D-alanine and D-proline, as a nitrogen source (Ngamskulrungroj et al., 2012). iCryptococcus model was able to use some amino acids (glutamine, glutamate, aspartate, glycine, proline, serine, alanine, threonine, arginine, isoleucine, valine, phenylalanine, methionine, cysteine, leucine, and lysine) and urea as nitrogen sources, in agreement with the literature information. Although experimental studies revealed that C. *neoformans* is able to consume creatinine and tryptophan as nitrogen sources, the pathogen is not able to use these two compounds in the iCryptococcus model.

*C. neoformans* is a facultative intracellular pathogen in the early stages of pulmonary infection. During the first 24 h after infection, the number of these cells peaks in alveolar macrophages (Feldmesser et al., 2001). The internal environment of macrophages is poor in nutrients and rich in stressors. Alternative carbon sources are preferred because the glucose used by the pathogen as a primary carbon source is limited in the macrophage environment (Barelle et al., 2006; Barkal et al., 2016). The environmental conditions in the early phase of the infection were here simulated and the results obtained by the iCryptococcus model were compared with those of experimental studies in the literature. To imitate the internal environment of macrophages in the lung, intracellular uptake of glucose was blocked in the iCryptococcus model, and the uptake of alternative carbon sources was turned on. Consistent with the results in the literature (Hu et al., 2008), the fluxes passing through the isocitrate lyase (ICL) and malate synthase (MALS) reactions catalyzed by enzymes encoded by the *CNH03280* and *CNH02910* genes in the glyoxylate cycle increased in the iCryptococcus model. Blocking the MALS reaction in the model negatively affected the growth of the organism on acetate, similar to the results of experimental studies (Hu et al., 2008). Aconitase (ACONT) reactions were also elevated and isocitrate dehydrogenase (ICDHxm) reactions were downregulated in *silico* model, as well as in experimental studies (Chen et al., 1997; Orner et al., 2019). In the iCryptococcus model, the gene *CNI03590* encodes phosphoenolpyruvate carboxykinase. This enzyme catalyzes the reaction responsible for phosphoenolpyruvate production in the gluconeogenesis pathway when glucose is limited. It converts oxaloacetate into phosphoenolpyruvate and carbon dioxide. In the iCryptococcus model, phosphoenolpyruvate carboxykinase (PPCK) catalyzed reaction had no flux when the carbon source was glucose. However, the flux through PPCK catalyzed reaction increased when infection conditions (e.g., zero glucose uptake rate, unlimited oxygen supply, carbon source such as acetate) were simulated (Barelle et al., 2006; Barkal et al., 2016). The fluxes through the glycolytic pathway, including glucose 6-phosphate isomerase and enolase catalyzed reactions, were also downregulated in the simulation by iCryptococcus model, consistent with the results given in the literature (Hu et al., 2008). Acetate utilization or production is potentially relevant to the pathogenesis of *C. neoformans* because it was one of the major metabolites present in infected tissue. Acetyl-CoA can be produced in a variety of ways, and activating acetate directly by acetyl-CoA synthase is one of them. The expression of the gene encoding the enzyme for the production of acetyl-CoA from acetate was elevated during pulmonary infection (Barelle et al., 2006). In the iCryptococcus model, this enzyme is encoded by the *CNA07740* gene and the flux of Acetyl-Coa synthetase (ACS) reaction increases under the condition of infection. The deletion of this reaction inhibits growth on acetate, ethanol and glycerol, in agreement with experimental results.

#### 3.2.1 Robustness analysis

**FIGURE 2:**
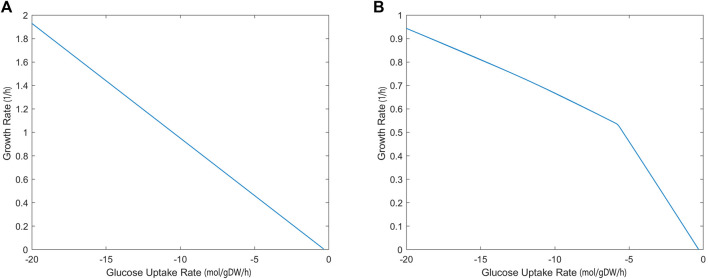
Glucose uptake rate (mmol/gDW/h)—growth rate (1/h) relationship without **(A)** and with **(B)** oxygen limitation, simulated by iCryptococcus model.

The growth rate calculated by the iCryptococcus model was 0.36/h when 4 mmol/gDW/h glucose was used as the carbon source (the amount of oxygen uptake was unlimited). As the amount of glucose increased, the growth rate increased linearly since the oxygen uptake was unlimited (Figure 2A). However, if the amount of oxygen in the environment was limited (17 mmol/gDW/h), the growth rate did not increase linearly, even if there was enough glucose (Figure 2B). Thus, at this point oxygen limited growth rate. Excess glucose could not be fully oxidized, thus the slope of the line decreased (Figure 2B). When the glucose uptake rate reached 18.5 mmol/gDW/h, the growth rate value remained around 0.9027 1/h.

#### 3.2.2 Shadow prices

**FIGURE 3:**
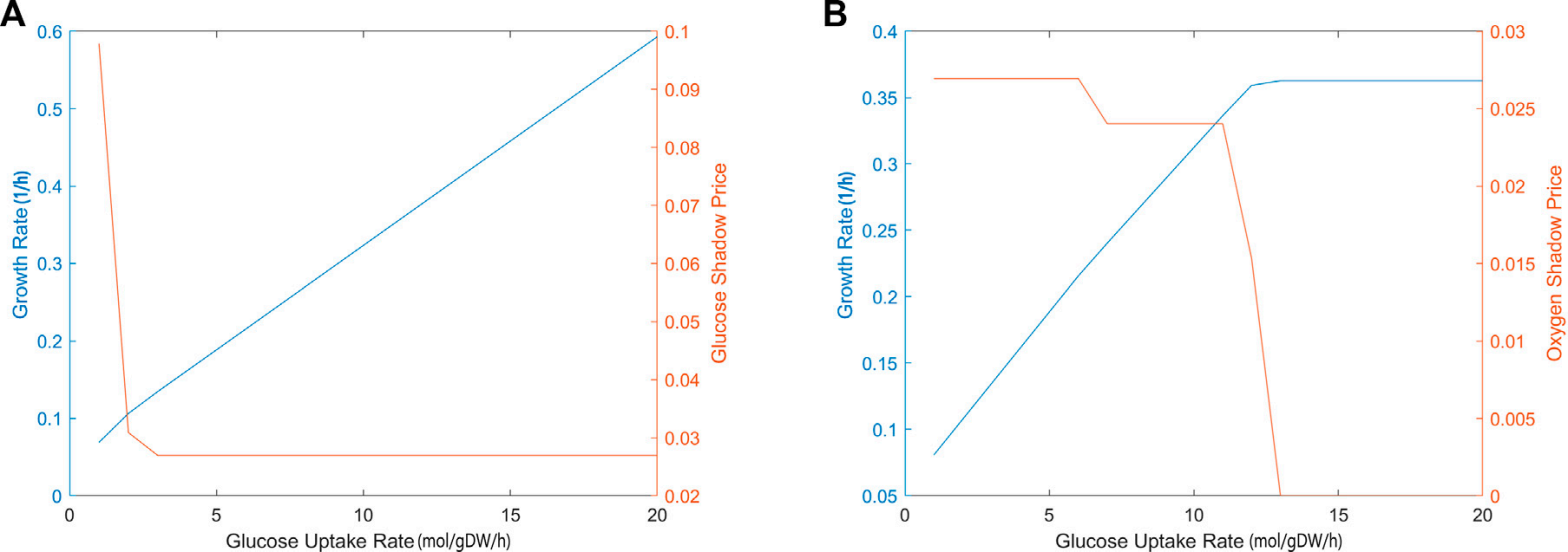
The calculated shadow prices of each solution based on glucose **(A)** and oxygen **(B)** uptake rates. The x-axis gives the flux of glucose uptake (EX glc D e) and oxygen uptake (EX o2 e). The y-axis on the left side shows the biomass objective function calculated depending on the change in glucose uptake. The y-axis on the right side gives the shadow prices of glucose and oxygen, respectively. During glucose uptake, the amount of oxygen was fixed at 4 mmol/gDW/h and during oxygen uptake, the amount of glucose was fixed at 4 mmol/gDW/h, respectively.

The sensitivity of biomass production to carbon source and oxygen availability was also evaluated through shadow price analysis, i.e., we determined how the addition of metabolites, glucose and oxygen, affects the biomass objective function. Figure 3 demonstrates that, as glucose uptake increased, the iCryptococcus model produced more biomass. While glucose uptake rate was in the range of 0–3 mmol/gDW/h, the shadow price of glucose decreased. As glucose uptake rate further increased, it remained constant. When glucose uptake was 3 mmol/gDW/h, the shadow price of glucose was 0.0280, and the growth rate was equal to 0.1399 1/h, i.e., if 1 mmol/gDW/h glucose is added, the biomass production will increase by 0.0280 (Figure 3A). For the calculation of oxygen shadow prices, the glucose uptake rate was fixed at 4 mmol/gDW/h. As the oxygen uptake rate increased in the range of 1–13 mmol/gDW/h, the biomass increased (Figure 3B). After the oxygen uptake rate of 12 mmol/gDW/h, the shadow price of oxygen decreased drastically.

### 3.3 Effect of virulence factors on pathogen metabolism

Growth ability at body temperature, phospholipase, urease, melanin production and capsule formation are important factors in the infectivity of *C. neoformans* (Buchanan., 1998; Hicks et al., 2004; Sabiiti and May., 2012; Huang et al., 2016). When the pathogen first infects humans through the respiratory tract, it is phagocytized by macrophages in the lung at the early infection stage. The intracellular structure of macrophages is poor in glucose, but there are alternative carbon sources in human body. In studies performed on *C. neoformans* samples isolated from macrophages, it was observed that the gene expression levels in glycolysis and TCA pathways decreased, whereas the gene expression levels in beta oxidation, glyoxylate cycle and gluconeogenesis pathways increased. Alternative pathways in the pathogen are possibly activated, which is in agreement with our simulation results mentioned in Section 3.2. Moreover, studies have also stated that the pathogen forms a thick capsule structure to prevent its digestion by the macrophage, and therefore the capsule and infectivity are related.

After the infection progresses through the circulation, *C. neoformans* gains access to vital organs such as the brain within the macrophages, and the capsule structure becomes almost non-existent with the decrease in stress conditions as observed in samples collected, probably due to the abundant glucose environment in the brain. A dynamic regulation takes place in the pathogen. Abundant glucose in the brain accelerates the proliferation and hence increases the virulence as an indirect effect. Carbon utilization is vital in terms of growth at body temperature. In order to elaborate on the effect of different carbon sources on biomass formation and virulence ability in later stages of infection, biomass production was calculated by supplying various carbon sources into the iCryptococcus model (Table 1). These results regarding growth and virulence are consistent with the literature. In the absence of glucose in the environment, the infectivity of the pathogen decreases, but stil prevails (Price et al., 2011). More research needs to be done to elaborate on the importance of different pathways and physical structures at different stages of infection.

**TABLE 1:** Biomass production on the carbon source.

Carbon source	Growth rate (1/h)
Glucose	0.36
Fructose	0.33
Acetate	0.03
Fumarate	0.15
Galactose	0.33
Glycerol	0.10
Succinate	0.17

In iCryptococcus model, the gene *CNC03080* encodes the enzyme pyruvate kinase. In cases where this gene was deleted, the flux in the biomass production reaction was reduced. Similarly, a decrease in viral activity was reported in *Cryptococcus* strains deficient in pyruvate kinase enzyme (Sabiiti and May., 2012). The genes *CND04180* and *CNM00920* in the iCryptococcus model encode the phospholipase B and lysophospholipase enzymes in the phospholipase class. These two enzymes are responsible for lipid degradation and the production of sn-glycero-3-phosphocholine. The simulations showed that, in the presence of a carbon source (glucose), there was a flux through the reaction responsible for sn-glycero-3-phosphocholine production, implying active reaction rate. Phospholipases affect membrane stabilization and lead to infection by suppressing the immune system (Sabiiti and May., 2012). Urease is the other important enzyme acting as a virulence factor (Cox et al., 2000). Urease enzyme is encoded by *CNH01900* in the iCryptococcus model. Depending on the increase in this enzyme activity in the model, the mitochondrial ATP production and the flux in the oxidative phosphorylation pathway increased.

### 3.4 Essentiality analyses

Essential genes found by single and double gene deletion analyses are important in terms of drug target potential, since biomass production is significantly reduced or stopped when these genes are at off status, and it is possible to stop the vital activities of the pathogen. However, in order to find drug targets without causing any side effects in humans, it is necessary to determine whether these genes have any ortholog in the human model (Recon3D).

#### 3.4.1 Single and double gene deletion phenotypes

All genes that contribute to the increase of the organism’s biomass production can also be detected by gene deletion analysis. In the iCryptococcus model, 153 of 649 genes were found as essential genes by single gene deletion analysis. Moreover, 202 of 649 genes, including 153 essential genes, were also found to affect biomass production. Of the 153 essential genes involved in various pathways, 58 of them do not have orthologs in the human model (Table 2).

**TABLE 2:** The distribution of essential genes that do not have an ortholog in the human model.

Genes	Pathway
CNB03110, CNC04470, CNF03720, CND03020	Steroid Biosynthesis
CNG02210, CNK03240, CNL04470, CNL05510, CNF01340	Oxidative Phosphorylation
CNA04370, CND03570, CNA06240	Arginine Biosynthesis
CNF01260	Purine Metabolism
CNA07120, CNG03730, CNL06550	Pyrimidine Metabolism
CNJ02910	Alanine, Aspartate and Glutamate Metabolism
CNA06290, CNC07110, CNI02030, CNA02450, CNJ02040, CNI02930, CNJ00410	Glycine, Serine and Threonine Metabolism
CND03580	Amino Sugar and Nucleotide Sugar Metabolism
CNA02570, CNN01460, CNF02480, CNH01530 CNH01520, CNA02270, CNH03010	Valine, Leucine and Isoleucine Biosynthesis and Degradation
CND01200, CNK00580, CND03850, CNG00170, CND06290	Lysine Biosynthesis
CNJ01640	Transport
CNM00100	Terpenoid Backbone Biosynthesis
CNB02610, CNH03390, CNN02320	Starch and Sucrose Metabolism
CNE00560, CNF02630, CNC06150	Riboflavin Metabolism
CND01510, CND06120, CNB01460, CNA07220, CNH01620, CNB03030	Histidine Metabolism
CNA07990	Phenylalanine Metabolism
CNB01990, CNH02650, CNI00560, CNA07880, CNF03410, CNM00820	Phenylalanine, Tyrosine and Tryptophan Biosynthesis
CNG04250	Glycerophospholipid Metabolism

Further on this issue, the double-gene deletion analysis and comparison with the human genes in the Recon3D model revealed eight essential gene pairs for *C. neoformans var. neoformans JEC21*. The essential gene pairs and reactions regulated by these genes are shown in Table 3. The discussion on these essential genes are given below along with metabolite and reaction deletion analyses, as these genes also encode the enzymes catalyzing the reactions involving essential metabolites.

**TABLE 3:** Essential gene pairs and reactions as a result of double gene deletion analysis.

Gene pair	Gene ID	Enzyme encoded	Reaction
1	CNH03280	Isocitrate lyase	ICL
	CNC04680		
		Threonine aldolase	THRA, THRA2
2	CNI03590	Phosphoenolpyruvate carboxykinase	PPCK
	CNK00280		
		Phosphoglycerate mutase	PGM
3	CNN00260	Sugar transporter	GLCt1, GALt2
	CNG01480		
		Hexose transport-related protein	GLCt2, FRUt2, MANt2
4	CNJ01880	Ammonium transporter	NH4t
	CNA02250		
			NH4t
5	CNF04620	Aminotran containing protein	AATA3, PHETA1
	CNB03180		
			AATA3, PHETA1
6	CNL06640	Phospho-2-dehydro-3-deoxyheptonate aldolase	DDPA, DDPAm
	CND05120		
			DDPA
7	CNG00040	Metabolite transporter	G3PCT
	CND01860		G3PCT
8	CNM00800	Amino acid transporter	CYSt2r, TYRt2r, GLNt2r, GLUt2r, ORNt2r, ASPt2r, ARGt2r, GLYt2r, ASNt2r, SERt2r, THRt2r, METt2r, LEUt2r, VALt2r, ILEt2r, LYSt2r, PROt2r
	CNM00800		

#### 3.4.2 Metabolite and reaction deletion phenotypes

The number of metabolites involved in at least three reactions in the iCryptococcus model is 379. As a result of the metabolite deletion analysis, 185 of 379 metabolites were found to be essential by the iCryptococcus model. 69 of these essential metabolites are currency metabolites. When the remaining 116 metabolites were compared with the human model (Recon3D), thirteen of these metabolites were found to be absent in the human model (Table 4).

**TABLE 4:** The distribution of essential metabolites not found in the human model.

Metabolite	Pathway
13BDglcn[c]	Starch and Sucrose Metabolism
2dda7p[c]	Phenylalanine, Tyrosine, and Tryptophan Biosynthesis
3c3hmp[c], 3c4mop[c]	Valine, Leucine, and Isoleucine Biosynthesis
4r5au[c]	Riboflavin Metabolism
aspsa[c]	Glycine, Serine, and Threonine Metabolism
chor[c]	Phenyalanine, Tyrosine, and Tryptophan Biosynthesis
epist[c], ergst[c], fecost[c]	Steroid Biosynthesis
pphn[c]	Phenylalanine, Tyrosine, and Tryptophan Biosynthesis
oxag[m]	Lysine Metabolism
chitin_[c]	Amino Sugar, and Nucleotide Sugar Metabolism

The iCryptococcus model has 71 essentials (58 genes and 13 metabolites) not found in the human model. In DrugBank database (Wishart., 2006), there are drugs targeting 16 of these essentials. The results of DrugBank research for the potential drug targets of the iCryptococcus model are given in Table 5. Some of these drugs are used to treat several other kinds of diseases. Thus, a drug repurposing process can be applied for approved or investigational drugs. Compared to creating a whole novel medicine for a certain use, this approach has a number of benefits.

**TABLE 5:** Drugbank result.

Target	Drugs	Organism
Ergosterol	Candicidin, Nystatin, Butoconazole, Amphotericin B, Natamycin, Clotrimazole	*Candida albicans*
Chorismate	Flavin mononucleotide	*Streptococcus pneumoniae, Helicobacter pylori*
1,3-β-glucan	Ibrexafungerp, Anidulafungin, Caspofungin, Micafungin	*Aspergillus niger*
CNA07880	5-O-phosphono-alpha-D-ribofuranosyl diphosphate	*Erwinia carotovora*
CNF01260	Flavin adenine dinucleotide, Azelaic acid	*Escherichia coli, Staphylococcus aureus*
CNN02320	Ibrexafungerp, Anidulafungin, Caspofungin, Micafungin	*Aspergillus niger*
CNH01520	alpha-Ketoisovalerate	*Mycobacterium tuberculosis*
CNA07120	Dihydroorotic Acid, Orotic acid, Lysine Nz-Carboxylic Acid, N-Carbamoylaspartic acid	*Escherichia coli*
CNG03730	5-O-phosphono-alpha-D-ribofuranosyl diphosphate, Orotic acid	*Salmonella typhimurium*
CNH02650	Flavin mononucleotide	*Streptococcus pneumoniae, Helicobacter pylori*
CNL06550	6-hydroxyuridine-5′-phosphate, 6-oxouridine 5′-phosphate	*Bacillus subtilis, Escherichia coli*
CNA02450	Nicotinamide adenine dinucleotide phosphate, (4s)-4-{[(2s)-2-Amino-3-Oxopropyl] Sulfanyl}-L-Homoserinate	*Haemophilus influenzae*
CNA02570	Triethylene glycol, Cocarboxylase	*Klebsiella pneumoniae*
CNA04310	3-Isopropylmalic Acid	*Thiobacillus ferrooxidans*
CNA07970	8-Hydroxy-2-oxa-bicyclo[3.3.1] non-6-ene-3,5-dicarboxylic acid	*Escherichia coli*
CNF02630	Dithioerythritol	*Mycobacterium tuberculosis*

**FIGURE 4:**
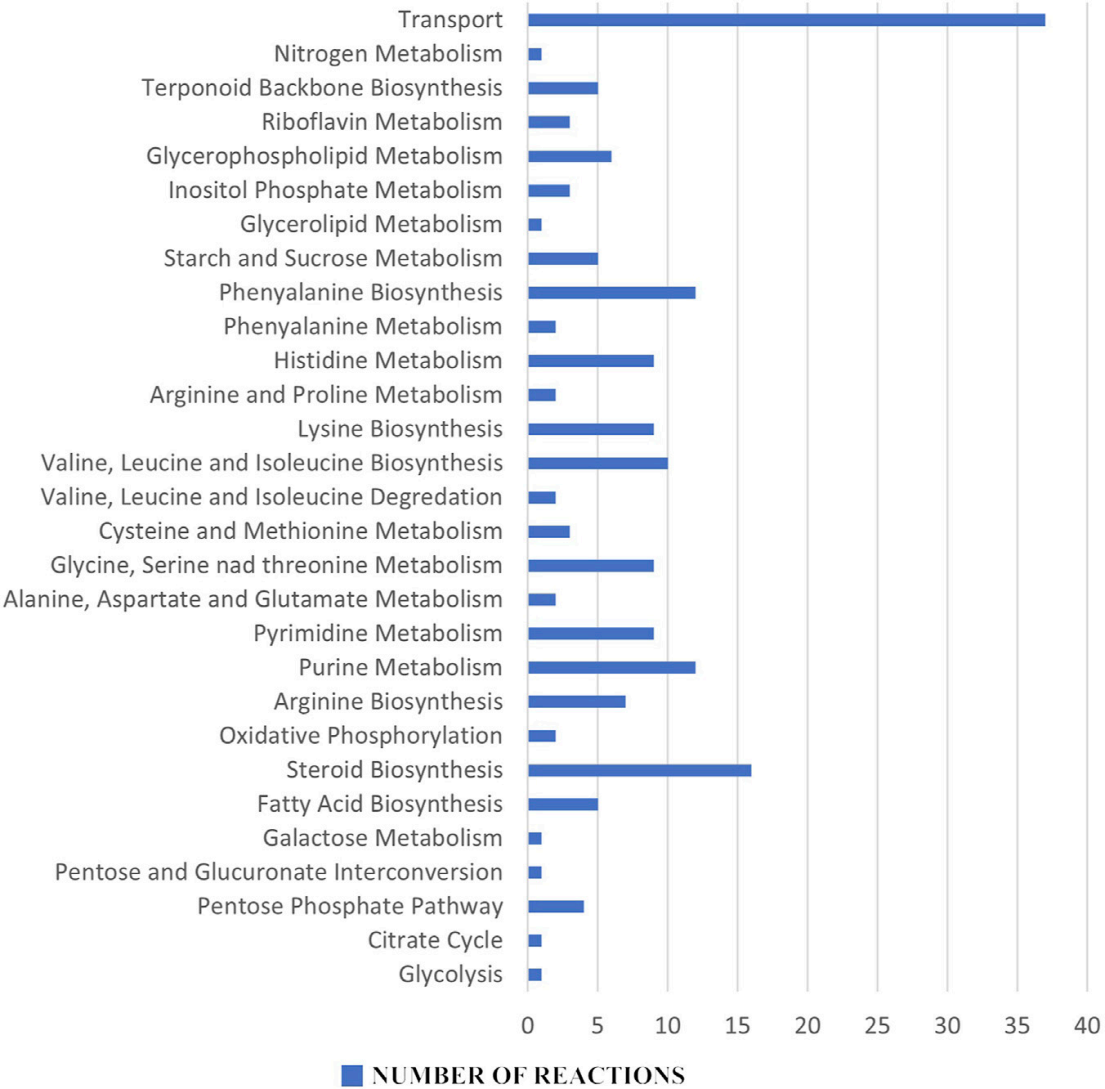
Essential reaction distribution: Among the 198 essential reactions, purine and pyrimidine metabolisms, amino acid metabolism, fatty acid biosynthesis and steroid biosynthesis all stand out as potential pharmacological targets for the next therapeutic approaches.

In the reaction essentiality analysis, 198 reactions were found to be essential out of a total of 1,270 reactions in the iCryptococcus model, irrespective of their involvement in the production of essential metabolites. Figure 4 shows the essential reaction distribution.

Metabolites and reactions essential for the vital activity of the pathogen, but not found in humans, are very important as potential drug targets. An inhibition in the production of essential metabolites and reactions in the pathogen metabolism will inhibit its proliferation and growth. Since these metabolites and reactions were not found in humans, the risk of any side effects to humans is eliminated. In this sense, 13 metabolites and 198 reactions (steroid metabolism, amino acid metabolism etc.,) found in the essentiality analysis have the potential to be drug targets for the therapeutic strategies to be developed.

## 4 Discussion

Studies have shown that *in silico* genome-scale models provide information about the complex processes occurring in metabolism and are important tools for many metabolic engineering methodologies. In this study, a genome-scale metabolic model specific to *C. neoformans*, which is responsible for more than hundreds of thousands of deaths annually worldwide was created in order to conduct detailed research on the metabolic processes of the pathogen by using computational systems biology approaches. The iCryptococcus model created includes the biochemical reactions involved in the synthesis of the main macromolecule components of the organism, along with the known virulence factors. Genome-scale metabolic models have a great advantage over traditional methods, with their stoichiometric consistency and system-level integration ability, discovering vital elements for the organism under different environmental conditions (Haggart et al., 2011; Sertbas and Ulgen 2020; Chen et al., 2022; Carey et al., 2022; Wendering and Nikoloski, 2022).

In the reconstructed iCryptococcus model, the genes, reactions and metabolites essential for growth were identified and the potential drug targets were unraveled. The iCryptococcus model contains 1,270 reactions in 57 pathways. Of these reactions, 239 are transport reactions and 110 are exchange reactions. There are 1,143 metabolites and 649 genes in this model. In addition, the model has two different objective functions, as the pathogen probably does not maximize the growth rate during infection. Moreover, virulence factors such as capsules are not vital for the growth of the organism *in vitro*. For this reason, the biomass reaction including the components for the synthesis of virulence factors was used in potential drug target investigations. Even if the growth rate of the pathogen does not reach the maximum level in the model with the complete biomass composition, for the purpose of the study it is important to determine the essential genes and reactions required for *in vivo* growth. The iCryptococcus model predicted that during the infection, carbon metabolism was actively used, and the utilization of carbon sources may affect the strength of infection, in agreement with the literature.

In the deletion (essentiality) analysis, 58 genes and 8 gene pairs of the 649 genes and 13 metabolites of the 1,143 total in the iCryptococcus model were found to be essential, and they do not have an ortholog in the human model. The pathways related to the metabolisms of starch and sucrose, amino acid, steroid etc. Appear to include these essential genes, metabolites and reactions, and can thus be considered as potential drug targets.

Chorismate (chor) is one of the 13 essential metabolites in the iCryptococcus model. It is an important biochemical intermediate found in plants and microorganisms. It is at the center of the biosynthesis of carboxylic aromatic compounds such as aromatic amino acid, vitamins E and K, ubiquinone. It is synthesized by the pathway called the shikimate pathway, which is involved in many bacteria and various fungal species. The presence of the pathway in many parasites and pathogens, but not in metazoa, makes it a prime drug target (Caspi et al., 2017). The genes (gene IDs: *CNH02650, CNF03410, CNI00560, and CNM00820*) that regulate chorismate-producing and consuming reactions in the iCryptococcus model were also found to be essential genes in gene essentiality analysis. Fortunately, these genes were not found in the human model. In this sense, chorismate can be a potential drug target, consistent with the study of Ziebart and colleagues (Ziebart et al., 2010) reporting that enzymes that utilize chorismate are important antimicrobial drug targets since they have a central role in survival and virulence.

2-dehydro-3-deoxy-D-arabino-heptonate 7-phosphate (2dda7p) is an essential metabolite in the iCryptococcus model. The first step of the biosynthesis of aromatic rings from carbohydrate precursors in microorganisms and plants begins with 3-deoxy-D-arabino-heptulosonate-7-phosphate. It is produced from phosphoenolpyruvate and D-erythrose-4-phosphate by an enzyme catalyzed by DAHP synthase. In the model, both this reaction and the genes *CND05120* and *CNL06640* regulating the reaction were found as a result of essentiality analysis. These results are consistent with those of Ducati and colleagues (Ducati et al., 2007), reporting that 3-deoxy-D-arabino-heptulosonate-7- phosphate synthase is an antimicrobial drug target since it is important for controlling carbon flow into the shikimate pathway.

L-aspartate 4-semialdehyde (aspsa) is a metabolite among the 13 essential metabolites. The reaction (reaction abbreviation: ASAD) producing the L-aspartate 4-semialdehyde is an essential reaction, and the gene (gene ID: *CNA02450*) that regulates this reaction is an essential gene in the iCryptococcus model. L-Aspartic-4-semialdehyde is an *a*-amino acid derivative of aspartate. It is an important intermediate of the aspartate pathway found in bacteria and plants in general. In this pathway, the biosynthesis of amino acids such as lysine, methionine and threonine from aspartate takes place. These results show that L-aspartate 4-semialdehyde can be a potential drug target, consistent with the study of Dahal and colleagues (Dahal et al., 2020) reporting that aspartate semialdehyde dehydrogenase is a drug target for antifungal drug development.

Chitin is one of the 13 essential metabolites in the iCryptococcus model. Along with glucan, chitin is an essential component of the organism’s cell wall structure. In addition, as a result of the deacetylation of chitin in the structure with the regulation of the *CND03580* gene, chitosan, another basic component of the cell wall structure, is formed. The cell wall structure, which consists of these three components, is necessary both for the growth of the organism and for its infectivity. In the results of the essential analysis, there is the *CND03580* gene, which regulates the reaction that plays a role in the consumption of chitin. These results show that chitin metabolites and *CND03580* gene can be a potential drug target, consistent with the literature (Li et al., 2019), and hence chitin synthase can be a drug target for antifungal drug development.

iCryptococcus model was able to find the targets of the main drug groups (polyenes, azoles and echinocandins) used for *C. neoformans* infections. In the iCryptococcus model, the essentialities of ergosterol metabolite and ergosterol production-related gene *CNC04470* are consistent with the drug target of the polyenes group drugs (Gray et al., 2012; Anderson et al., 2014). Ergosterol is a sterol found in fungi, similar to cholesterol in mammalian cells, and plays an important role in cell membrane integrity. The essentialities of fecosterol and episterol metabolites and fecosterol production gene *CNB03100* are consistent with the drug target of the azole group drugs (Anderson et al., 2014; Allen et al., 2015; Kathiravan et al., 2012). Fecosterol and episterol are not present in cholesterol biosynthesis in mammals, but are involved in the first step of fungal-specific biosynthesis. The essentialities of 1,3-*β*-glucan metabolite and 1,3-*β*-glucan production-related gene *CNN02320* are consistent with the drug target of the echinocandins group drugs (Feldmesser., 2001). Alpha and beta-glucan structures constitute nearly 90% of the cell wall biomass of *C. neoformans*. The cell wall structure has an important effect on the survival of the organism. In addition, a deformation in the structure of the cell wall causes a decrease in virulence.

Seventy one key components (58 genes and 13 metabolites) unique to the iCryptococcus model were also searched in DrugBank, and 16 of these were found to be targeted by several drugs for different diseases. Thus, a drug repurposing procedure can be used with authorized or experimental drugs. This strategy has a lot of advantages over developing an entire unique drug specifically for a given need.

We were able to confirm the expected virulence factors and growth patterns as well as identify new drug targets in this fungal pathogen using our high-quality model, iCryptococcus. This first-of-its-kind genome-scale metabolic model of *C. neoformans* can be improved upon using laboratory and biochemical studies, omics data, and other sources of information. This model will open the door to a better understanding of the metabolism of this human pathogen, its interactions, and its prominence. In order to narrow the range of viable flux phenotypes, the integration of omics data, including transcriptomics and metabolomics, may be used as additional constraints. One may also incorporate thermodynamic limitations. Such a technique allows for modeling condition-specific metabolism combining data from reaction thermodynamics and transcriptomics/metabolomics, and provides a thermodynamically feasible metabolic model. Despite some errors due to limited coverage of the whole genome of an organism, genome-scale metabolic reconstructions have shown to be quite effective at identifying new drug targets, and if such a model is reconstructed, drug targets can be forecast, that is followed by the discovery of effective medications and the experimental validation of these targets.

## Data Availability

The original contributions presented in the study are included in the article/supplementary material, further inquiries can be directed to the corresponding author.
